# Factors associated with early failure of the femoral neck system (FNS) in patients with femoral neck fractures

**DOI:** 10.1186/s12891-023-06994-7

**Published:** 2023-11-27

**Authors:** L Chen, JB Jiang, H Ma, X Duan, JL Chen

**Affiliations:** 1 Department of Orthopedic Surgery, West China Hospital, Sichuan University, Chengdu, China; 2 Trauma Center, West China Hospital, Sichuan University, Chengdu, China

**Keywords:** Femoral neck system, Femoral neck fractures, Internal fixation, Early failure

## Abstract

**Background:**

Femoral neck system (FNS) is a new type of internal fixation system which has been widely used for treating femoral neck fractures (FNFs).Compared with other internal fixation methods, FNS is minimally invasive and stable, and often achieves satisfactory short-term efficacy.Early failure of FNS (EFFNS) is not uncommon, however, there are few literatures and reports on factors associated with EFFNS.This study aimed to survey the prevalence and risk factors of EFFNS.

**Methods:**

We retrospectively analysed 62 patients with FNFs and underwent FNS fixation between 2019 and 2021. Demographic data, clinical characteristics, radiographic features and treatment process were described. Multifactor logistic regression analysis was used to analyse the different influencing factors.

**Results:**

Out of the 62 FNFs patients, 10 patients (16.1%) developed EFFNS, including 6 cases of severe femoral neck shortening, 2 cases of screw-out, 1 case of avascular necrosis of the femoral head and 1 case of nonunion. In the failure group, all patients were younger than 65 years old, which was significantly higher than 59.6% in the healing group (*P* = 0.012). There were no significant differences in sex(*P* = 0.490), BMI (*P* = 0.709), injured side (*P* = 0.312), injury mechanism (*P* = 0.617), reduction method(*P* = 0.570),femoral neck-shaft angle(*P* = 0.545), Pauwels classification (*P* = 0.564) and Garden classification (*P* = 0.195). Moreover, we not found that Garden classification (*P* = 0.464) and age (*P* = 0.128) were statistically significant risk factors for EFFNS at multivariate analysis.

**Conclusion:**

In this study, sex, BMI, injury side, injury mechanism, reduction method, Pauwels angle, femoral neck-shift angle, Pauwels classification and Garden classification were excluded as EFFNS risk factors. Moreover, our study demonstrated that age and Garden classification were not significant risk factors at multivariate analysis.

**Trial registration:**

ChiCTR, ChiCTR2100051360. Registered on 21 September, 2021. https://www.chictr.org.cn/index.aspx.

## Introduction

As the population ages, femoral neck fractures (FNFs) are becoming a common injury in middle-aged and elderly people, accounting for approximately 54% of hip fractures [1, 2]. Various approaches to internal fixation such as cannulated compression screws (CCSs) [3], sliding hip screws [4], Hansson pin system [5] and dynamic hip screws (DHSs) [6] have been used for the treatment of FNFs.

However,failure of internal fixation and functional loss after surgery are not uncommon and often lead to rehospitalization or even more serious consequences [7, 8]. Different studies have reported that 7–22% of patients receive secondary operations, mostly due to complications, such as avascular necrosis of the femoral head (ANFH), nonunion, severe femoral neck shortening (SFNS), and screw-out [9, 10]. To reduce postoperative complications, researchers have been committed to innovation of internal fixation methods and devices. In recent years, researchers have developed the femoral neck system (FNS)(DePuy Synthes, USA), which consists of four parts: a barrel plate, a blade, an antirotation screw and a locking screw. The FNS is a novel internal fixation system featuring anti-rotation, anti-sliding, and anti-shearing functions for the treatment of FNFs.

Several studies have reported comparative efficacy between FNS and other internal fixation approaches, suggesting that the short-term efficacy of FNS is satisfactory [11–13]. In addition, the literature also reported that early failure of the FNS (EFFNS) occurred after internal fixation of FNFs.The previous literature suggested that there were many risk factors for the failure of internal fixation of FNFs, among which the Garden classification was an important one that has been widely discussed [14, 15]. However, there is a paucity of literature looking at the factors associated with EFFNS in patients with FNFs. Therefore, we assume that the Garden classification is one of the risk factors for EFFNS. In this study, we aimed to investigate the prevalence of EFFNS among patients with FNFs, verify the validity of the hypothesis and identify other risk factors for EFFNS in patients with FNFs.

## Materials and methods

### Study design

This study was performed at a level-I orthopedic trauma centre from October 2019 to March 2021. A series of patients with a diagnosis of femoral neck fracture were treated operatively using the FNS technique.We conducted a retrospective study of the factors associated with early failure of the femoral neck system (FNS) in these patients with femoral neck fractures.

### Inclusion and exclusive criteria

All subjects considered in this study had to be admitted to our trauma unit in the selected period and had undergone operative intervention FNS. The AO/OTA (Albeitgemainshaft fur Osteosynthesisfrag/Orthopaedic Trauma Association) classification was 31-B [16]. Age was 18 years old or older, with no sex limitation. Only patients capable of walking independently or with aid before the trauma and who did not present serious impairment of consciousness were included in the study. Exclusion criteria included pathological fractures, < 6 months of follow-up, postoperative infection, pre-existing femoral head necrosis, developmental dysplasia of the hip, severe hip arthritis, multiple traumatic injuries, and any risk factors that induce ANFH or fracture nonunion, such as long-term hormone application or smoking, and alcohol abuse.

### Surgical techniques, postoperative treatment, and rehabilitation programs

All surgeries were performed at our institution by senior orthopedic surgeons. The surgical procedures were based on standard protocols for FNS [17]. Firstly, we insert a 2.5 mm guide pin to maintain fracture reduction.Then, insert a 130° guide pin along the femoral neck as the central guide. Under C-arm fluoroscopy, adjust the insertion point and angle of the guide to ensure it located in the center of the femoral neck in both the anteroposterior and lateral positions. Measure the length and choose the proper implant. Expand the hole along the center guide and insert the bolt-and-plate assembly into the femoral head.Finally, position the anti-rotation screw and locking screw in appropriate location. Antibiotic intravenous prophylaxis was administered with cefazolin 30 min preoperatively and 24 h postoperatively. Patients with stable fractures (Garden type I and II) were allowed partial weight-bearing on the second day postoperatively. Meanwhile, patients with unstable fractures (Garden type III and IV) were allowed to undergo isometric contraction exercises of the quadriceps of the femoris and active and passive flexion and extension training of the ankle joint to reduce oedema of the lower extremities. Low-molecular weight heparin (0.2–0.4 ml, according to the weight of patients) was routinely injected until discharge to prevent deep vein thrombosis. All patients were required to return to the outpatient department for follow-up at 1, 3, and 6 months postoperatively. Partial weight-bearing and full weight-bearing exercises were gradually increased according to the rate of fracture healing on X-ray radiography performed at each visit.

### Patient assessment

Two external and independent investigators not involved in the patients' treatment were responsible for data collection. Demographic data, medical history, surgery data and radiographic data at presentation were collected. The following demographic information was recorded: patient age, sex, and body mass index (BMI). BMI (kg/m^2^) was calculated by dividing weight (kg) by the square of height (m).Medical history included injury mechanism and injury side.The mechanism of injury that can easily cause femoral neck fracture was defined into four categories: fall from standing, road accident, fall from height and others. Surgical information included injury-to-surgery interval and fracture reduction methods (open reduction or closed reduction). Standard preoperative and postoperative anteroposterior and lateral radiographs of the femoral neck were used to evaluate the radiographic features of the patients [18]. The radiographs were analyzed by two independent observers who knew nothing about the outcomes [19]. All FNFs were radiographically classified by the Garden classification [20] and the Pauwels system [21]. The Pauwels angle was defined as the angle between the fracture line and the horizontal plane and measured before surgery [22]. The femoral neck-shaft angle was assessed as the angle between the longitudinal femoral shaft axis and the femoral head-neck axis and measured immediately after surgery [23].

Standard radiographic and clinical follow-up were scheduled at 1, 3, and 6 months postoperatively and continued until fracture healing or until a main complication leading to EFFNS. Complications were defined as ANFH,SFNS, nonunion, and screw-out. ANFH was defined as a cortical collapse of the femoral head, while SFNS was defined as a degree of shortening of the femoral neck greater than 10 mm. The shortening degree was calculated by analysis of the displacement of the screw normalized to the length of the barrel for FNS and described previously by Vazquez *et al* [13]. Nonunion was defined as the absence of radiographic and clinical signs of fracture healing within 6 months and screw-out as a cut-out of the bolt-and-plate or the antirotation screw or loosening of the locking screw. The cohort was then divided into two groups, depending on the complications leading to failure of the internal fixation or healing of the fracture: failure group and healing group.

### Statistical analysis

Continuous variables are expressed as mean ± standard deviation, and categorical variables are expressed as absolute values and percentages. All data were analysed using the Wilcoxon rank sum test (Mann–Whitney U test). Differences in continuous variables were tested using the two-tailed Student’s t-test, and differences in categorical variables were assessed using the Pearson *χ*^*2*^ test or Fisher’s exact test where appropriate. Multifactor logistic regression analysis was used to analyse the different influencing factors. Odds ratios and 95% confidence intervals were calculated. All statistical analyses were carried out using SPSS version 21.0 statistical software (IBM Corp. Released 2021. IBM SPSS Statistics for Windows, Version 28.0. Armonk, NY: IBM Corp).A *P* value < 0.05 was regarded as statistically significant.

## Results

### Patients’ characteristics

**Fig. 1:**
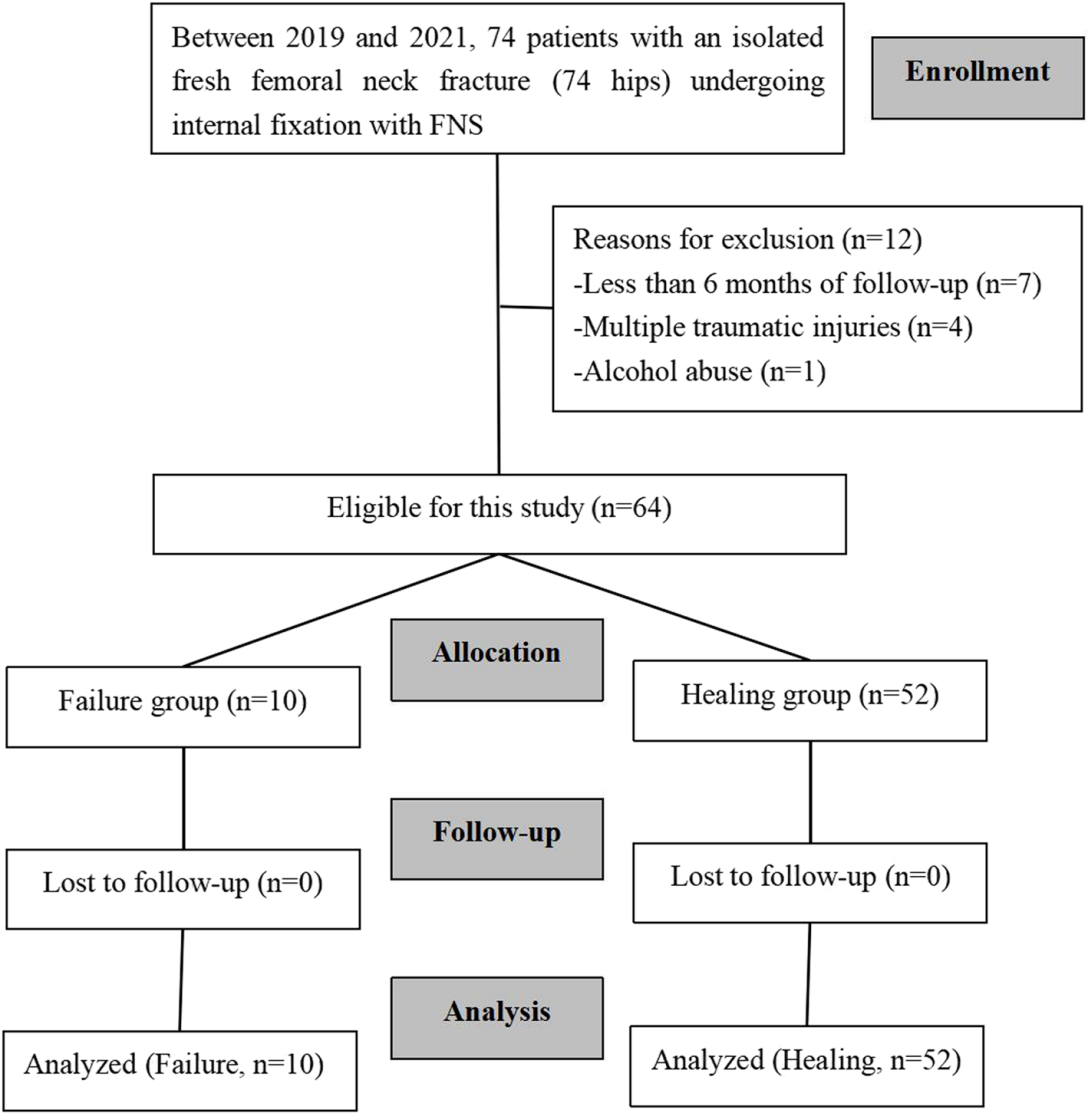
Flow diagram showing femoral neck fracture patients’ recruitment,allocation and analysis

**Fig. 2:**
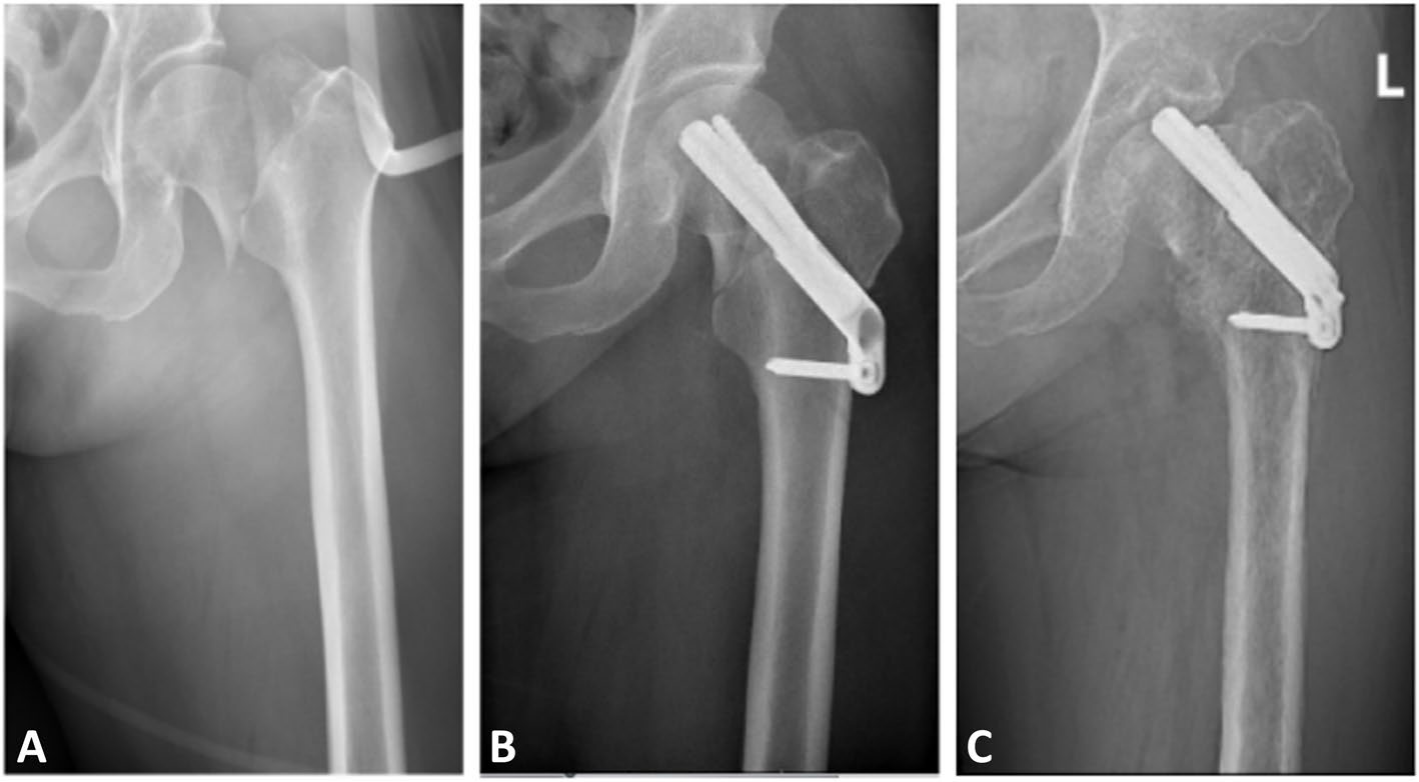
Preoperative anteroposterior radiograph of a left femoral neck fracture (Garden IV) in a 57-year-old woman (**A**). Anteroposterior radiograph at 2-month follow-up showing excellent healing and no evidence of femoral head necrosis (**B**). Anteroposterior radiograph at 6-month postoperatively showing screw-out and femoral head necrosis (**C**)

Between 2019 and 2021, 74 consecutive patients with a diagnosis of FNF underwent FNS fixation at our hospital. Twelve of them were excluded based on inclusion and exclusion criteria, leaving 62 patients with a mean age of 56 years (range 20–93 years) who met the inclusion criteria and were analyzed(two groups: failure group [*n* = 10] versus healing group [*n* = 52]) (Fig. 1). Patients were followed up for a minimum of 6 months to a maximum of 15 months. The average BMI was 22.7 (range 12.0–30.1). There were 33 right hips and 29 left hips. The sex distribution was 50:50. All patients had successful surgeries. There were 10 cases of EFFNS with 6-month follow-up, accounting for 16.1% of all patients. Ten patients showed pain or dysfunction during the rehabilitation exercise during the follow-up period and came to seek medical treatment. After their physical examination and imaging was reviewed, cases of EFFNS were identified, including 6 cases of SFNS, 1 case of ANFH, 1 case of nonunion, and 2 cases of screw-out (Fig. 2). In terms of treatment options, a total of 5 patients received THA, including 1 case of ANFH, 2 cases of screw-out and 2 cases of SFNS. The other 5 patients (4 cases of SFNS and 1 case of nonunion) chose to continue observation because the symptoms were tolerable.

There were no significant differences in sex (*P* = 0.490), BMI (*P* = 0.709), injury mechanism (*P* = 0.617), injury-to-surgery interval (*P* = 0.466) or injury side (*P* = 0.312). However, in the failure group, all patients were younger than 65 years old. The proportion was significantly higher than 59.6% of the healing group (*P* = 0.012) (Table 1).

**Table 1:** Demographic details of patients

Variable	Failure group (*n *= 10)	Healing group (*n* = 52)	*P* value
**Age, no. (%)**			0.012^*a^
≥ 65 years old	0 (0)	21 (40.4%)	
< 65 years old	10 (100%)	31 (59.6%)	
**Gender, no. (%)**			0.490^b^
Male	4 (40%)	27 (51.9%)	
Female	6 (60%)	25 (48.1%)	
**Injury side, no. (%)**			0.312^a^
Left side	3 (30%)	26 (50%)	
Right side	7 (70%)	26 (50%)	
**BMI (kg/m**^**2**^**), mean ± SD**	23.50 ± 3.04	22.59 ± 2.91	0.709^c^
**Injury-to-surgery interval (days), mean ± SD**	9.70 ± 5.79	7.69 ± 3.56	0.466^c^
**Injury mechanism, no. (%)**			0.617^d^
Fall from standing	8 (80%)	37 (71.2%)	
Road accident	1 (10%)	7 (13.5%)	
Fall from height	0 (0)	6 (11.5%)	
Others	1 (10%)	2 (3.8%)	

*BMI* Body mass index, *no.* Number of patients, *SD* Standard deviation

^*^P < 0.05

^a^Analyzed using Fisher’s exact test

^b^Analyzed using chi-square test

^c^Analyzed using independent sample t test

^d^Analyzed using Mann-Whitnery U test

### Comparison of surgery and radiographic data

There were no significant differences in reduction methods (*P* = 0.570), Pauwels angle (*P* = 0.542), or femoral neck-shaft angle (*P* = 0.545). No significant differences were observed in terms of Garden classification (*P* = 0.195) or Pauwels classification (*P* = 0.564) (Table 2).

**Table 2:** Comparison of surgery and radiographic data between the two groups

Variable	Failure group (*n* = 10)	Healing group (*n* = 52)	*P* value
**Reduction methods, no. (%)**			0.570^a^
Close reduction	8 (80%)	43 (82.7%)	
Open reduction	2 (20%)	9 (17.3%)	
**Pauwels angle (°), mean ± SD**	59.90 ± 16.38	58.33 ± 15.98	0.542^b^
**Neck-shaft angle (°), mean ± SD**	138.50 ± 5.95	138.02 ± 6.03	0.545^b^
**Garden classification, no. (%)**			0.195^c^
I	1 (10%)	3 (5.8%)	
II	0 (0)	10 (19.2%)	
III	2 (20%)	15 (28.8%)	
IV	7 (70%)	24 (46.2%)	
**Pauwels classification, no. (%)**			0.564^c^
I	1 (10%)	4 (7.7%)	
II	1 (10%)	12 (23.1%)	
III	8 (80%)	36 (69.2%)	

*no.* Number of patients, *SD* Standard deviation

^a^Analyzed using Fisher’s exact test

^b^Analyzed using independent sample t test

^c^Analyzed using Mann-Whitnery U test

### Multifactor logistic regression analysis

Multifactor logistic regression analysis indicated that Garden classification (*P* = 0.464) and age (*P* = 0.128) were not statistically significant risk factors for the EFFNS (Table 3).

**Table 3:** Logistic regression analysis of factors for early failure of Femoral Neck System

Variable	B	S.E	Wald	*P* value	Exp (B)/OR	95% CI	
						Lower	Upper
**Age**	0.050	0.033	2.315	0.128	1.051	0.986	1.121
**Garden classification**	1.077	1.470	0.537	0.464	2.937	0.165	52.371
**Constant**	-5.021	2.868	3.065	0.080	0.007		

## Discussion

Surgical treatment of FNFs mainly comprises closed or open reduction and internal fixation and primary arthroplasty. The internal fixation implants are CCSs, SHSs, DHSs and Hansson pin system, while primary arthroplasty includes total hip arthroplasty (THA) and hemiarthroplasty [24]. Many factors including displacement of the femoral neck, presence of hip osteoarthritis, age, reduction quality, and stable internal fixation affect the surgeon's decision on the surgery method [17].

The ideal implant is considered as a conduct with the characteristics of strong fixation of fractures, prevention of femoral neck shortening, and avoidance of tilting and rotation of the femoral head [25]. The FNS is a newly developed femoral neck internal fixation device in recent years. It contains a bolt, an anti-rotation screw and a femoral lateral plate. This plate has 1 hole or 2 holes for the standard 5.0 mm locking screw. After assembly, the FNS forms a stable structure with an angle of 130° in the femoral neck and femoral shaft. This stable structure combines the advantages of angular stability and minimally invasive surgical techniques, and allows the bolt and anti-rotation screw to slide together in the plate barrel to dynamically compress the fractured end, similar to a DHS. However, the surgical incision for an FNS is smaller than that for a DHS, thereby reducing soft tissue damage and protecting the blood supply. Therefore, FNS is considered the next generation of internal fixation devices for the treatment of FNFs [26]. It combines many advantages, including providing sufficient angular stability, reducing blood supply damage, dynamic compression and anti-rotation.

A few advantages of the FNS are due to its biomechanical characteristics. Fan et al. indicated that the internal fixation stress of FNS was higher than that of CCS in finite element analysis, which is approximately 1.6–3.0 times that of CCS in Pauwels III fractures at 50°, 60°, and 70° [27]. Especially at 70°, the displacement of the double-hole FNS was the smallest in the various groups. A biomechanical loading test conducted by Stoffel et al. evaluated the performance of FNS in comparison with DHS and CCS [28]. The experiment increased at a rate of 0.1 N/cycle until the termination criteria were achieved. The study found that cycles until 15 mm leg shortening and 15 mm femoral neck shortening in FNS were significantly higher than those in CCS. Similarly, Schopper et al. evaluated the biomechanical performance between FNS and Hansson pin systems in models of Pauwels II FNFs [29]. They indicated that the angular stability of the FNS provided superior resistance against varus deformation and performed in a less sensitive way to variations in implant placement.

Several comparative studies have reported the clinical outcomes between FNS and various internal fixations, indicating that the short-term efficacy of FNS is satisfactory [11–13, 17, 26]. However, FNS related complications, such as SFNS, ANFH, nonunion or delayed healing, and screw-out, have also been reported in these literature.

A total of 6 patients developed SFNS in this study. One of the characteristics of the FNS is dynamic compression. The precollapsed insertion allows the anti-rotation screw and bolt to slide in the maximum 20 mm packaging to meet femoral neck shortening during fracture healing. Similar to a DHS, femoral neck shortening after FNS placement is also a common phenomenon. The principle of fracture site compression utilized by surgical constructs may promote healing. However, SFNS is associated with worse patient-reported outcomes and objective functional measures. Most studies defined SFNS as a shortening of 10 mm or longer in length. Both the retrospective FAITH trial [30] and the prospective SHOC trial [31] showed that SFNS after internal fixation was associated with inferior functional outcomes. Similarly, Zlowodzki et al. found differences in scores related to the degree of shortening, indicating worse functional outcomes with a greater degree of shortening [32]. Therefore, in this study, half of SFNS patients chose to receive THA, and their function was partially restored after surgery.

In this study, all EFFNSs existed in young and middle-aged patients (under 65 years old). The general treatment strategy for FNFs is widely considered to be that internal fixation is more suitable for young and middle-aged patients, while THA is more suitable for elderly patients with poor physical condition and bone quality. However, this strategy did not form a consensus. A meta-analysis by Xu et al. reviewed 2065 patients with FNFs from 17 case–control studies and found no association between age and osteonecrosis of the femoral head [33]. However, another meta-analysis by Slobogean et al. reviewed 1558 cases of FNFs from 41 studies, indicating that the high total incidence of ANFH in patients under 60 years old was 14.3%, and nonunion was 9.3% [9]. In addition, for elderly patients, the best functional results could be achieved in patients with a well-healed femoral neck without ANFH after urgent reduction and internal fixation of displaced FNFs [34]. Therefore, patient selection and surgical skill were important factors influencing clinical outcomes. We suggest that future studies emphasize the importance of surgical indications in the young and middle-aged patient populations. Furthermore, the promotion of surgical skills is key for avoiding postoperative complications and EFFNS.

Garden classification was an important parameter when considering surgery in FNF patients. There were a few studies supporting the Garden classification as one of the risk factors for ANFH after internal fixation of FNFs [14, 33, 35, 36]. In this study, displaced fractures (Garden III and IV) in the failure group accounted for 90%, while in the healing group, they accounted for only 75%. Although there was no statistical significance, EFFNS may be associated with the high proportion of displaced fractures. However, after regression analysis, we did not find support for Garden classification as a risk factor for EFFNS. The reason may be the short follow-up time (6 months) of this study, and the short-term efficacy of FNS was satisfactory.The good anti-rotation, anti-sliding and anti-shear function of FNS, as well as the reduction of blood supply injury, may reduce the occurrence of early complications of displaced FNF.And long-term clinical outcomes after FNS fixation need to be determined with longer follow-up.All cases during follow-up did not routinely performed MRI examinations but only X-rays.However, the manifestation of ANFH can only be clearly seen on X-rays of Ficat III-IV patients [19], so some ANFH patients in Ficat I-II may be overlooked during outpatient follow-up.Therefore, we recommend that regular review of MRI for early detection and treatment of ANFH.

In addition, we could not find the relationship between EFFNS and sex, BMI, injured side, injury mechanism, reduction method, Pauwels angle, femoral neck-shaft angle, or Pauwels classification.

This study had some limitations. Firstly, it was a single-centre study. Therefore, there must have been some selective bias. Secondly, the small number of enrolled patients can not provide enough information and may result in sample bias. Thirdly,this study did not investigate other possible risk factors, such as weight-bearing time, anatomical classification, posterior tilt, preoperative bone quality and bone mineral density, and length of the screws, that may have significantly affected the prognosis of the patients. Hence, future studies need to be conducted to clarify these issues.

## Conclusion

In summary, our study suggests that age and Garden classification were not found significant risk factors at multivariate analysis. Moreover, we cannot determine whether age and Garden classification are independent risk factors for EFFNS in the treatment of FNFs. In this study, sex, BMI, injured side, injury mechanism, reduction method, Pauwels angle, femoral neck-shaft angle, and Pauwels classification were ruled out as correlative risk factors for EFFNS.

## Data Availability

The original data of this study are available from the corresponding author for reasonable request.

## Abbreviations

ANFH: Avascular necrosis of the femoral head
BMI: Body mass index
CCS: Cannulated compression screws
DHS: Dynamic hip screws
EFFNS: Early failure of the femoral neck system
FNFs: Femoral neck fractures
FNS: Femoral neck system
MRI: Magnetic resonance imaging
SFNS: Severe femoral neck shortening
THA: Total hip arthroplasty
